# C9orf72’s Interaction with Rab GTPases—Modulation of Membrane Traffic and Autophagy

**DOI:** 10.3389/fncel.2016.00228

**Published:** 2016-10-07

**Authors:** Bor L. Tang

**Affiliations:** ^1^Department of Biochemistry, Yong Loo Lin School of Medicine, National University of SingaporeSingapore, Singapore; ^2^NUS Graduate School for Integrative Sciences and Engineering, National University of SingaporeSingapore, Singapore

**Keywords:** C9orf72, Rab, membrane trafficking, autophagy, ALS

## Abstract

Hexanucleotide repeat expansion in an intron of Chromosome 9 open reading frame 72 (*C9orf72*) is the most common genetic cause of Amyotrophic Lateral Sclerosis (ALS) and Frontotemporal Dementia (FTD). While functional haploinsufficiency of C9orf72 resulting from the mutation may play a role in ALS/FTD, the actual cellular role of the protein has been unclear. Recent findings have now shown that C9orf72 physically and functionally interacts with multiple members of the Rab small GTPases family, consequently exerting important influences on cellular membrane traffic and the process of autophagy. Loss of C9orf72 impairs endocytosis in neuronal cell lines, and attenuated autophagosome formation. Interestingly, C9orf72 could influence autophagy both as part of a Guanine nucleotide exchange factor (GEF) complex, or as a Rab effector that facilitates transport of the Unc-51-like Autophagy Activating Kinase 1 (Ulk1) autophagy initiation complex. The cellular function of C9orf72 is discussed in the light of these recent findings.

## Introduction

Amyotrophic Lateral Sclerosis (ALS) is a progressive, fatal neurodegenerative disease affecting motor neurons (Kiernan et al., 2011; Riva et al., 2016). About 10% of ALS cases have a hereditary component, and more than 30 genes have now been identified for familial ALS (Iguchi et al., 2013; Renton et al., 2014). Frontotemporal Dementia (FTD), on the other hand, is a clinical syndrome of neuropsychiatric disorders associated with degeneration of the prefrontal and anterior temporal cortex (Neary et al., 2005). These two seemingly clinically disparate neurodegenerative diseases are genetically linked (Andersen, 2013; Bennion Callister and Pickering-Brown, 2014), and have some common pathological features in terms of components of neuronal cytoplasmic inclusion, or stress granules (Bentmann et al., 2013). Mutations in genes encoding RNA binding proteins such as the TAR DNA binding protein of 43 kDa (TDP-43; Sreedharan et al., 2008) and Fused-in-sarcoma (FUS; Kwiatkowski et al., 2009) causes familial ALS, and these mutant proteins are found both in neuronal cytoplasmic inclusions of ALS patients and those with the most common pathological phenotype of FTD, namely frontotemporal lobar degeneration (FTLD; Neumann et al., 2006; Mackenzie et al., 2010). The clinically classified ALS and FTD may indeed be part of a clinicopathological spectrum of diseases with overlapping etiology and pathology (Andersen, 2013; Thomas et al., 2013; Devenney et al., 2015). A prominent genetic linkage point between ALS and FTD has been mapped to chromosome 9p (Vance et al., 2006; Pearson et al., 2011). In 2011, an ALS-FTD causative mutation was identified in the form of a GGGGCC hexanucleotide repeat expansion (from a typical 5–10 repeats to hundreds or more) in an intron of the gene Chromosome 9 open reading frame 72 (*C9orf72*; DeJesus-Hernandez et al., 2011; Renton et al., 2011). This mutation turns out to be the most common cause of ALS/FTD in multiple cohorts worldwide (DeJesus-Hernandez et al., 2011; Majounie et al., 2012; Rohrer et al., 2015).

*C9orf72* orthologs are found in most vertebrates and some invertebrates like *C. elegans*, and phylogenetic analysis indicated that it is likely present in the common ancestor of eukaryotes (Levine et al., 2013a). In humans, *C9orf72* has three alternatively spliced transcripts, predicted to produce two polypeptide isoforms (of 481 and 222 amino acid residues in length, respectively). The most prominent feature of the protein is the presence of a “Differentially expressed in normal and neoplastic cells” (DENN) domain (Zhang et al., 2012; Levine et al., 2013a). The DENN domain is a type of Longin/Roadblock domain fold that are interacting platforms present in several classes of proteins in the eukaryotic membrane trafficking machinery (Levine et al., 2013b), and several DENN domain containing proteins are verified Rab Guanine nucleotide exchange factors (GEFs; Marat et al., 2011). This structural feature brings to fore a possible function for this hitherto uncharacterized pathological factor, namely, as modulators of Rab GTPase activities.

Understanding the cellular role of C9orf72 may shed light on ALS/FTD pathology, as an etiological consequence resulting from a loss of function of the protein could be entertained. The G-rich hexanucleotide repeat extension may contribute to genetic haploinsufficiency by forming stable G-quadruplex structures that disrupt transcription (Fratta et al., 2012) or promote hypermethylation of the gene locus, thereby attenuating C9orf72 expression (Xi et al., 2013). Loss or targeted silencing of C9orf72 orthologs in *C. elegans* (Therrien et al., 2013) and zebrafish (Ciura et al., 2013) have both resulted in morphological and functional motor neuron deficits. Interestingly, however, depletion of C9orf72 in mouse brain by about half via intracerebroventricular injection of antisense oligonucleotides did not result in any discernible motor or behavioral impairment (Lagier-Tourenne et al., 2013), neither was any neuronal defects observed in neural-specific mice knockouts (nestin-Cre excision of floxed *C9orf72*, Koppers et al., 2015). In fact, C9orf72-null mice developed normally and aged mice do not suffer from motor neuron disease (O’Rourke et al., 2016), but have other phenotypes that may well be relevant to C9orf72’s recently deciphered roles in membrane trafficking and autophagy.

Mutant C9orf72 may cause disease pathology through several other mechanisms, which including gain-of-function type toxicity resulting from dysregulation of RNA metabolism and dipeptides generated by Repeat-associated non-ATG (RAN) translation of the hexanucleotide expansion (Ash et al., 2013; Mori et al., 2013; May et al., 2014; Mizielinska et al., 2014). These mechanisms have been discussed in recent excellent reviews (Mizielinska and Isaacs, 2014; Gitler and Tsuiji, 2016; Todd and Petrucelli, 2016). The focus here shall instead be on recent findings of how C9orf72 may influence cellular membrane traffic and autophagy though Rab GTPases, and these are discussed elaborated in the paragraphs below.

## C9orf72’s Interaction with Rab GTPases and Its Influence on Membrane Trafficking

C9orf72 has diverse subcellular distribution. It could be found in both cytoplasmic and nuclear compartments (Farg et al., 2014; Sellier et al., 2016), and is also secreted extracellularly, with the latter likely occurring via some form of unconventional exocytosis (Chua et al., 2012; Malhotra, 2013; Ponpuak et al., 2015). Perhaps the only clue to its function is its DENN domain (Levine et al., 2013a), which is shared by a number of Rab GEFs (Marat et al., 2011; Zhang et al., 2012). Together with the observation that C9orf72 decorates some intracellular membranes, its DENN domain makes it likely that it may modulate membrane traffic via regulating Rab activities. Atkin and colleagues tested this directly by co-immunoprecipitation and GFP-Trap in human and mouse neural cell lines and primary neurons, and showed that Rabs 1, 5, 7 and 11 are amongst those proteins associated with C9orf72 (Farg et al., 2014). C9orf72 also exhibited a good degree of membrane co-localization with the endocytic Rabs 7 and 11. An indication that C9orf72 may be involved in endocytic transport was provided by the observation that its silencing affected the endocytic trafficking of Shiga toxin B and BDNF-bound TrkB to varying degrees (Farg et al., 2014). However, a mechanistic understanding of C9orf72’s association with these endocytic Rabs is still lacking, and whether their guanine nucleotide binding status is indeed modified by C9orf72 remains unclear.

In another recent report, the role of C9orf72 functioning as part of a GEF complex to a different set of Rabs has been revealed (Sellier et al., 2016). The authors found, by affinity tag purification and mass spectrometric sequencing, a set of cellular associates of C9orf72, amongst which are Rab8A and Rab39B. Two other interacting proteins are of particular interest. One of these, WD-40 repeat 41 (WDR41), is a WD-40 repeat domain containing protein with unknown function (Stein et al., 2011). The other, Smith-Magenis Syndrome chromosome region candidate 8 (SMCR8), is also a DENN domain containing protein (Zhang et al., 2012). SMCR8 and WDR41 directly interact with C9orf72, and these form a tripartite complex which has prominent affinity for Rab8A and Rab39B (and also interacts more weakly with a few other Rabs). The complex also exhibits GEF activity that could be measured *in vitro* towards both these Rabs, but has no such activity towards another Rab that it does not appear to interact with. This interaction between C9orf72 and SMCR8/WDR41 has also been reported by another laboratory (Sullivan et al., 2016). Two observations made with regards to the C9orf72-SMRC8-WDR41 complex are noteworthy. Firstly, the interaction between the complex and the Rabs are mostly dependent on SMRC8, which on its own could associate with several Rabs. However, when bound to WDR41 and C9orf72, its affinity is more prominent towards Rab8A and Rab39B. Secondly, recombinant C9orf72 alone has little or no GEF activity towards these Rabs, and it appears to act as such only in complex with SMCR8 and WDR1.

Does the new C9orf72-containing complex and their GEF activity towards Rab8A and Rab39B inform us of C9orf72’s role in membrane trafficking? Rab8A is an ubiquitously expressed Rab with a well-known role in modulating polarized plasma membrane transport in neurons (Huber et al., 1993) and non-neuronal (Peränen et al., 1996) cells. It is apparently critical for transport to the primary cilium (Yoshimura et al., 2007) and is also known to regulate neuronal trafficking and dendritic transport of both the ionotrophic AMPA receptor (Brown et al., 2007) and metabotropic glutamate receptor (Esseltine et al., 2012). Rab39B, on the other hand, is a brain-enriched Rab whose mutations have been implicated in X-linked intellectual and developmental disability (Giannandrea et al., 2010; Andersen et al., 2014) and early-onset Parkinson’s disease (PD; Wilson et al., 2014; Lesage et al., 2015; Mata et al., 2015). Reminiscent of Rab8A, Rab39B has also been recently shown to specifically regulate dendritic trafficking of AMPA receptor subunit GluA2 through its effector Protein interacting with C kinase 1 (PICK1; Mignogna et al., 2015). Although it remains to be investigated, it is conceivable that the C9orf72-containing GEF complex has a role in dendritic trafficking of glutamate receptors and other cargos.

## C9orf72’s Modulation of Autophagy Through Its Interaction with Rab GTPases

The process of macroautophagy intersects with vesicular membrane trafficking through Rab GTPases (Chua et al., 2011; Ao et al., 2014; Szatmári and Sass, 2014), and C9orf72’s interaction with Rabs indicate that it could also modulate autophagic flow, a critical disease modifying process in many neurodegenerative diseases that result from impaired clearance of toxic protein/RNA aggregates (Jain and Ganesh, 2016). ALS/FTD are likewise characterized by impairment of aggrephagy, the autophagosome mediated clearance of ubiquitinated proteins (Thomas et al., 2013). In this regard, C9orf72 was indeed shown to localize to autophagosome-like structures that were positive for the autophagosome marker Microtubule-associated protein 1A/1B-light chain 3 (LC3; Farg et al., 2014). Sellier et al. (2016) have now showed that silencing of C9orf72 expression in neurons attenuated the formation of mTOR inhibitor-induced LC3B-positive autophagosomes. A reduction in C9orf72 expression also promoted TDP-43 aggregation, and synergizes with Ataxin-2 intermediate-length polyglutamine expansions (polyQ), a known genetic risk of ALS (Bonini and Gitler, 2011; Wang et al., 2014), in promoting neuronal death. A number of the C9orf72-associating proteins identified by Sellier et al. (2016) have established connections with autophagy. The most obvious of these is the autophagy adaptor Sequestosome 1/p62, whose mutations have been associated with ALS/FTD (Rubino et al., 2012). Another recent report has shown that even an apparently conservative mutation of p63, namely L341V, could be disease causing as the mutant has significantly reduced binding affinity to LC3B (Goode et al., 2016).

**Figure 1:**
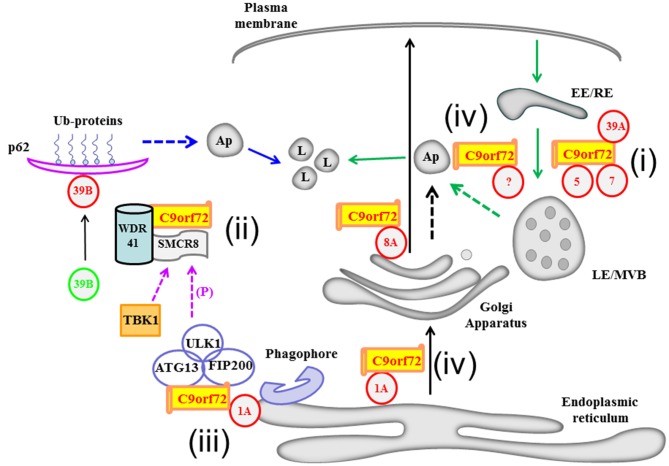
**A schematic diagram illustrating Chromosome 9 open reading frame 72 (C9orf72)’s recently deciphered interaction with various Rab GTPases and its possible roles in membrane traffic and autophagy.** C9orf72’s interaction with Rab1A and Rab8A may influence transport along the early and post-Golgi exocytic pathways, respectively (black arrows). Rabs are depicted in red circles with their respective numbers. C9orf72’s functional interactions with the Rabs described in the text are summarized (i to iv): i. C9orf72’s interaction with Rab5, Rab7 and Rab39A may affect endocytic transport (green arrows). ii. Acting as part of the Smith-Magenis Syndrome chromosome region candidate 8 (SMCR8) and WD-40 repeat 41 (WDR41) containing complex, C9orf72 is part of a Guanine nucleotide exchange factor (GEF) complex which activates Rab39B (denoted as a change from green to red). Activated Rab39B’s subsequent engagements of effectors molecules, such as p62, acts in autophagic clearance of ubiquitinated (Ub) substrates. iii. On the other hand, C9orf72 could bridge the ULK1 autophagy initiating complex with Rab1A as an effector of the latter, thus delivering the ULK1 complex to the phagophore. iv. Autophagosomes may have multiple origins, including the membranes of the ER (blue dotted arrow), Golgi apparatus/TGN (black dotted arrow) and the endosomal membranes (green dotted arrow), and C9orf72 may have a role to play in autophagosome generation from these compartments. There is a complex crosstalk between the C9orf72 interacting proteins, for example ULK1 phosphorylates SMCR8, and the autophagy-initiating complex may thus also promote downstream engagement of the autophagy scaffolding protein p62. LE/MVB, late endosome/multivesicular body; EE/RE, early endosome/recycling endosome; Ap, autophagosome.

Pertaining to the Rabs, Rab8B, a close paralogs of Rab8A that has overlapping functions with the latter in polarized transport (Sato et al., 2014), is known to act through the innate immunity regulator, TRAF family member-associated NF-κB activator (TANK)-binding kinase 1 (TBK1; Weidberg and Elazar, 2011), in autophagic elimination of mycobacteria in macrophages (Pilli et al., 2012). The C9orf72-SMRC8-WDR41 complex does not appear to bind TBK1 directly, but was shown to associate with TANK and other TBK1 adaptors. SMRC8 appears to be a substrate of TBK1, and the latter’s phosphorylation of SMRC8 is important for autophagy regulation. Phosphorylation-dead mutants of SMCR8 were unable to rescue autophagy defects resulting from its initial silencing, while phosphomimetic mutants rescued defects resulting from TBK1 depletion. The notion that SMCR8 phosphorylation is important for autophagy induction was also attested to by the finding that it is a substrate of the autophagy initiating kinase, Unc-51-like kinase 1 (ULK1)/ATG1 (Noda and Fujioka, 2015; Xu et al., 2015). Both the Rab8 isoforms could therefore be part of a complex autophagy-modulating network that involves C9orf72 (see Figure 1). Interestingly though, overexpression of either wild-type or constitutively active Rab8A did not rescue the defect resulting from C9orf72 silencing, which suggest that the role of both these Rabs in autophagy modulation is complex and non-linear.

On the other hand, expression of a constitutively active Rab39B did rescue the autophagy defect resulting from decreased expression of C9orf72 (Sellier et al., 2016). As mentioned above, Rab39B mutations were implicated in X-linked mental disability with developmental defects and early-onset PD. Although shown to be localized to Golgi-derived vesicles (Giannandrea et al., 2010) and implicated in exocytic neuritogenesis and glutamate receptor transport (Mignogna et al., 2015), the cellular function of Rab39B is still largely unclear. A close paralog of Rab39B, Rab39A, which is more ubiquitously expressed (but at much lower levels in the brain than Rab39B) and known to be localized to the late endocytic compartments (Becker et al., 2009; Seto et al., 2011; Gambarte Tudela et al., 2015), has been previously implicated in negative regulation of bacterial lipopolysaccharide-induced autophagy in macrophages (Seto et al., 2013). Based on the above, it does not appear that the role and activity of Rab39A could be equated with the yet undefined activity of Rab39B in autophagy in neurons. A vaguely relevant pathological feature of PD resulting from Rab39B mutation is α-synuclein pathology, which is relevant to autophagy impairment (Xilouri et al., 2016). One plausible mode of action of Rab39B was suggested by the authors (Ciura et al., 2016; Sellier et al., 2016). Activated by the C9orf72-SMRC8-WDR41 GEF complex, Rab39B then engages p62 to facilitate autophagic clearance of aggregated proteins, such as Ataxin2 polyQ (Figure 1). However, more work is clearly needed to decipher Rab39B’s roll in C9orf72 modulation of autophagy.

Another recent finding has added to the complexity of the picture, and implicated Rab1A in C9orf72’s autophagy modulating function (Webster et al., 2016). The authors showed by a proximity ligation assay and *in vitro* binding experiments that C9orf72 interacts with all three members of the mammalian autophagy initiation complex, namely Ulk1, ATG13 and the FAK family kinase-interacting protein of 200 kDa (FIP2000; Lin and Hurley, 2016). Although C9orf72 does not affect ULK1 activation resulting from mTOR inhibition, its loss appeared to affect ULK1 complex translocation to the phagophore by Rab1A (Lynch-Day et al., 2010; Wang et al., 2013). Indeed, a yeast 2-hybrid interaction screen identified Rab1A as an interacting partner of C9orf72. C9orf72 binds preferentially to GTP-loaded active Rab1A, and is thus a Rab1A effector, rather than regulator. As a downstream effector of Rab1A, C9orf72 appears to mediate the interaction between the GTPase and the Ulk1 complex, acting as a translocation adaptor to bring these to the sites of autophagosome formation.

## C9orf72 and Rabs in a Complex Network of Autophagy Modulation

Taking the above findings into consideration, C9orf72 thus has apparent roles in modulating both cellular membrane traffic and autophagy. If the C9orf72-containing GEF complex influenced the function of Rab8A, it should regulate polarized dendritic transport in neurons, and transport to neuronal primary cilia. Furthermore, depending on what it interacts with downstream of Rab1A, it may have a role in the early exocytic pathway. On the other hand, C9orf72’s co-immunoprecipitation with Rab5, Rab7 and Rab11 points towards a role in endocytic transport and recycling. This remains to be confirmed by further analysis.

Pertaining to autophagy, C9orf72 appears to have a dual function, one in inducing autophagosome formation (acting downstream of Rab1A; Webster et al., 2016), and another in directing aggregated protein clearance through p62 (acting upstream of Rab39B; Ciura et al., 2016; Sellier et al., 2016). There are clear points of crosstalk between these two C9orf72-centric nodes. Ulk1 phosphorylates and therefore modulates SMRC8’s activity, and could potentially couple the activation of the initiation complex and phagosome formation to a downstream p62-mediated capture of autophagosomal cargo (see Figure 1). This notion, while perhaps over-simplified, would explain the autophagy defects associated with loss of C9orf72. It should also be noted that the C9orf72-SMRC8-WDR41 GEF complex may also regulate autophagy via other Rabs other than Rab39B. Amongst the Rabs which showed a weaker association with the complex, Rab12 is known to facilitate autophagosome trafficking (Xu et al., 2015). Furthermore, close paralogs of Rab32 and Rab33A, namely Rab38 (Hirota and Tanaka, 2009) and Rab33B (Itoh et al., 2008), have all been implicated in some aspects of autophagy.

How do these deciphered roles and activities in membrane trafficking and autophagy in neuronal cells translate to its role during development and ALS/FTD pathology? *C9orf72*−/− mice have enlarged lymph nodes and spleens, with p62 and LC3 levels clearly elevated in the C9orf72-deficient spleens (O’Rourke et al., 2016). Cells of myeloid lineage, such as macrophages and microglia, exhibited an age-dependent pro-inflammatory response, with defects in endolysosomal transport, lysosomal function and likely lysosomal degradation of autophagosomes. These phenotypes are somewhat in line with C9orf72’s modulation of exocytic/endocytic membrane traffic and autophagic clearance, as discussed above. In a broad sense, the anticipated loss of C9orf72 variant expression and function in human mutations should have some contribution towards the disease state in ALS/FTD. However, this may act “silently” alongside the more prominent and progressive pathological features of RNA foci, and the more acutely toxic (Kanekura et al., 2016), intercellular transmissible Gly-Ala dipeptide polymers (Chang et al., 2016).

## Future Perspectives

In the paragraphs above, I have outlined and discussed recent findings on C9orf72’s interactions with Rab GTPases that point towards its role in the modulation of membrane traffic and autophagy. Clearly we are only beginning to get a glimpse of what C9orf72 does in cells, and more details of its complex interactions with Rabs and their regulators/effectors will likely be further revealed. Future investigations on the cellular roles and functional consequences of the putative interactions between C9orf72 and Rabs (including those other than Rab1A, Rab8 and Rab39B) should be pursued. Understanding of how the mutant hexanucleotide expansion of C9orf72 affect neuronal membrane traffic and autophagy would benefit from analyses using appropriate cellular and *in vivo* models, including gene editing based knock-in. In particular, whether and how the key processes of membrane dynamics are perturbed by mutant C9orf72 in the relevant neuron types affected in ALS/FTD should be of urgent interest. New knowledge that we get from these studies will undoubtedly facilitate interpretation of disease pathology, and would hopefully in due course inform intervention strategies.

## Note Added in Proof

While the manuscript was in its final stage of review, Chen and colleagues reported a C9ORF72/SMCR8/WDR41-containing complex that also contain ATG101 (Yang, M., Liang, C., Swaminathan, K., Herrlinger, S., Lai, F., Shiekhattar, R., and Chen, J.-F. (2016). A C9ORF72/SMCR8-containing complex regulates ULK1 and plays a dual role in autophagy. Sci. Adv. 2(9): e1601167). The C9orf72 complex has Rab39B GEF activity, and also regulates Ulk1 levels as well as autophagic activity.

## Author Contributions

BLT conceived and wrote the article.

## Conflict of Interest Statement

The author declares that the research was conducted in the absence of any commercial or financial relationships that could be construed as a potential conflict of interest.

## References

[B2] Andersen P. M. (2013). ALS and FTD: two sides of the same coin? Lancet Neurol. 12, 937–938. 10.1016/s1474-4422(13)70218-7 24011654

[B1] Andersen E. F. Baldwin E. E. Ellingwood S. Smith R. Lamb A. N. (2014). Xq28 duplication overlapping the int22h-1/int22h-2 region and including RAB39B and CLIC2 in a family with intellectual and developmental disability. Am. J. Med. Genet. A. 164A, 1795–1801. 10.1002/ajmg.a.36524 24700761

[B3] Ao X. Zou L. Wu Y. (2014). Regulation of autophagy by the Rab GTPase network. Cell Death Differ. 21, 348–358. 10.1038/cdd.2013.187 24440914 PMC3921601

[B4] Ash P. E. A. Bieniek K. F. Gendron T. F. Caulfield T. Lin W. L. Dejesus-Hernandez M.. (2013). Unconventional translation of C9ORF72 GGGGCC expansion generates insoluble polypeptides specific to c9FTD/ALS. Neuron 77, 639–646. 10.1016/j.neuron.2013.02.004 23415312 PMC3593233

[B5] Becker C. E. Creagh E. M. O’Neill L. A. J. (2009). Rab39A binds caspase-1 and is required for caspase-1-dependent interleukin-1β secretion. J. Biol. Chem. 284, 34531–34537. 10.1074/jbc.m109.046102 19833722 PMC2787314

[B6] Bennion Callister J. Pickering-Brown S. M. (2014). Pathogenesis/genetics of frontotemporal dementia and how it relates to ALS. Exp. Neurol. 262, 84–90. 10.1016/j.expneurol.2014.06.001 24915640 PMC4221591

[B7] Bentmann E. Haass C. Dormann D. (2013). Stress granules in neurodegeneration—lessons learnt from TAR DNA binding protein of 43 kDa and fused in sarcoma. FEBS J. 280, 4348–4370. 10.1111/febs.12287 23587065

[B8] Bonini N. M. Gitler A. D. (2011). Model organisms reveal insight into human neurodegenerative disease: ataxin-2 intermediate-length polyglutamine expansions are a risk factor for ALS. J. Mol. Neurosci. 45, 676–683. 10.1007/s12031-011-9548-9 21660502 PMC3207127

[B9] Brown T. C. Correia S. S. Petrok C. N. Esteban J. A. (2007). Functional compartmentalization of endosomal trafficking for the synaptic delivery of AMPA receptors during long-term potentiation. J. Neurosci. 27, 13311–13315. 10.1523/jneurosci.4258-07.2007 18045925 PMC6673392

[B10] Chang Y. J. Jeng U. S. Chiang Y. L. Hwang I. S. Chen Y. R. (2016). The glycine-alanine dipeptide repeat from C9orf72 hexanucleotide expansions forms toxic amyloids possessing cell-to-cell transmission properties. J. Biol. Chem. 291, 4903–4911. 10.1074/jbc.M115.694273 26769963 PMC4777828

[B11] Chua C. E. L. Gan B. Q. Tang B. L. (2011). Involvement of members of the Rab family and related small GTPases in autophagosome formation and maturation. Cell. Mol. Life Sci. 68, 3349–3358. 10.1007/s00018-011-0748-9 21687989 PMC11114630

[B12] Chua C. E. L. Lim Y. S. Lee M. G. Tang B. L. (2012). Non-classical membrane trafficking processes galore. J. Cell. Physiol. 227, 3722–3730. 10.1002/jcp.24082 22378347

[B13] Ciura S. Lattante S. Le Ber I. Latouche M. Tostivint H. Brice A.. (2013). Loss of function of C9orf72 causes motor deficits in a zebrafish model of amyotrophic lateral sclerosis. Ann. Neurol. 74, 180–187. 10.1002/ana.23946 23720273

[B14] Ciura S. Sellier C. Campanari M. L. Charlet-Berguerand N. Kabashi E. (2016). The most prevalent genetic cause of ALS-FTD, C9orf72 synergizes the toxicity of ATXN2 intermediate polyglutamine repeats through the autophagy pathway. Autophagy 12, 1406–1408. 10.1080/15548627.2016.1189070 27245636 PMC4968221

[B15] DeJesus-Hernandez M. Mackenzie I. R. Boeve B. F. Boxer A. L. Baker M. Rutherford N. J.. (2011). Expanded GGGGCC hexanucleotide repeat in noncoding region of C9ORF72 causes chromosome 9p-linked FTD and ALS. Neuron 72, 245–256. 10.1016/j.neuron.2011.09.011 21944778 PMC3202986

[B16] Devenney E. Vucic S. Hodges J. R. Kiernan M. C. (2015). Motor neuron disease-frontotemporal dementia: a clinical continuum. Expert Rev. Neurother. 15, 509–522. 10.1586/14737175.2015.1034108 25865485

[B17] Esseltine J. L. Ribeiro F. M. Ferguson S. S. G. (2012). Rab8 modulates metabotropic glutamate receptor subtype 1 intracellular trafficking and signaling in a protein kinase C-dependent manner. J. Neurosci. 32, 16933–16942. 10.1523/JNEUROSCI.0625-12.2012 23175844 PMC6621761

[B18] Farg M. A. Sundaramoorthy V. Sultana J. M. Yang S. Atkinson R. A. K. Levina V.. (2014). C9ORF72, implicated in amytrophic lateral sclerosis and frontotemporal dementia, regulates endosomal trafficking. Hum. Mol. Genet. 23, 3579–3595. 10.1093/hmg/ddu068 24549040 PMC4049310

[B19] Fratta P. Mizielinska S. Nicoll A. J. Zloh M. Fisher E. M. C. Parkinson G.. (2012). C9orf72 hexanucleotide repeat associated with amyotrophic lateral sclerosis and frontotemporal dementia forms RNA G-quadruplexes. Sci. Rep. 2:1016. 10.1038/srep01016 23264878 PMC3527825

[B20] Gambarte Tudela J. Capmany A. Romao M. Quintero C. Miserey-Lenkei S. Raposo G.. (2015). The late endocytic Rab39A GTPase regulates the interaction between multivesicular bodies and chlamydial inclusions. J. Cell Sci. 128, 3068–3081. 10.1242/jcs.170092 26163492

[B21] Giannandrea M. Bianchi V. Mignogna M. L. Sirri A. Carrabino S. D’Elia E.. (2010). Mutations in the small GTPase gene RAB39B are responsible for X-linked mental retardation associated with autism, epilepsy and macrocephaly. Am. J. Hum. Genet. 86, 185–195. 10.1016/j.ajhg.2010.01.011 20159109 PMC2820185

[B22] Gitler A. D. Tsuiji H. (2016). There has been an awakening: emerging mechanisms of C9orf72 mutations in FTD/ALS. Brain Res. 1647, 19–29. 10.1016/j.brainres.2016.04.004 27059391 PMC5003651

[B23] Goode A. Butler K. Long J. Cavey J. Scott D. Shaw B.. (2016). Defective recognition of LC3B by mutant SQSTM1/p62 implicates impairment of autophagy as a pathogenic mechanism in ALS-FTLD. Autophagy 12, 1094–1104. 10.1080/15548627.2016.1170257 27158844 PMC4990988

[B24] Hirota Y. Tanaka Y. (2009). A small GTPase, human Rab32, is required for the formation of autophagic vacuoles under basal conditions. Cell. Mol. Life Sci. 66, 2913–2932. 10.1007/s00018-009-0080-9 19593531 PMC11115675

[B25] Huber L. A. Pimplikar S. Parton R. G. Virta H. Zerial M. Simons K. (1993). Rab8, a small GTPase involved in vesicular traffic between the TGN and the basolateral plasma membrane. J. Cell Biol. 123, 35–45. 10.1083/jcb.123.1.35 8408203 PMC2119815

[B26] Iguchi Y. Katsuno M. Ikenaka K. Ishigaki S. Sobue G. (2013). Amyotrophic lateral sclerosis: an update on recent genetic insights. J. Neurol. 260, 2917–2927. 10.1007/s00415-013-7112-y 24085347

[B27] Itoh T. Fujita N. Kanno E. Yamamoto A. Yoshimori T. Fukuda M. (2008). Golgi-resident small GTPase Rab33B interacts with Atg16L and modulates autophagosome formation. Mol. Biol. Cell 19, 2916–2925. 10.1091/mbc.E07-12-1231 18448665 PMC2441679

[B28] Jain N. Ganesh S. (2016). Emerging nexus between RAB GTPases, autophagy and neurodegeneration. Autophagy 12, 900–904. 10.1080/15548627.2016.1147673 26985808 PMC4854548

[B29] Kanekura K. Yagi T. Cammack A. J. Mahadevan J. Kuroda M. Harms M. B.. (2016). Poly-dipeptides encoded by the C9ORF72 repeats block global protein translation. Hum. Mol. Genet. 25, 1803–1813. 10.1093/hmg/ddw052 26931465 PMC4986334

[B30] Kiernan M. C. Vucic S. Cheah B. C. Turner M. R. Eisen A. Hardiman O.. (2011). Amyotrophic lateral sclerosis. Lancet 377, 942–955. 10.1016/S0140-6736(10)61156-7 21296405

[B31] Koppers M. Blokhuis A. M. Westeneng H. J. Terpstra M. L. Zundel C. A. C. Vieira de Sá R.. (2015). C9orf72 ablation in mice does not cause motor neuron degeneration or motor deficits. Ann. Neurol. 78, 426–438. 10.1002/ana.24453 26044557 PMC4744979

[B32] Kwiatkowski T. J. Bosco D. A. Leclerc A. L. Tamrazian E. Vanderburg C. R. Russ C.. (2009). Mutations in the FUS/TLS gene on chromosome 16 cause familial amyotrophic lateral sclerosis. Science 323, 1205–1208. 10.1126/science.1166066 19251627

[B33] Lagier-Tourenne C. Baughn M. Rigo F. Sun S. Liu P. Li H. R.. (2013). Targeted degradation of sense and antisense C9orf72 RNA foci as therapy for ALS and frontotemporal degeneration. Proc. Natl. Acad. Sci. U S A 110, E4530–E4539. 10.1073/pnas.1318835110 24170860 PMC3839752

[B34] Lesage S. Bras J. Cormier-Dequaire F. Condroyer C. Nicolas A. Darwent L.. (2015). Loss-of-function mutations in RAB39B are associated with typical early-onset Parkinson disease. Neurol. Genet. 1:e9. 10.1212/nxg.0000000000000009 27066548 PMC4821081

[B35] Levine T. P. Daniels R. D. Gatta A. T. Wong L. H. Hayes M. J. (2013a). The product of C9orf72, a gene strongly implicated in neurodegeneration, is structurally related to DENN Rab-GEFs. Bioinformatics 29, 499–503. 10.1093/bioinformatics/bts725 23329412 PMC3570213

[B36] Levine T. P. Daniels R. D. Wong L. H. Gatta A. T. Gerondopoulos A. Barr F. A. (2013b). Discovery of new longin and roadblock domains that form platforms for small GTPases in ragulator and TRAPP-II. Small GTPases 4, 62–69. 10.4161/sgtp.24262 23511850 PMC3747258

[B37] Lin M. G. Hurley J. H. (2016). Structure and function of the ULK1 complex in autophagy. Curr. Opin. Cell Biol. 39, 61–68. 10.1016/j.ceb.2016.02.010 26921696 PMC4828305

[B38] Lynch-Day M. A. Bhandari D. Menon S. Huang J. Cai H. Bartholomew C. R.. (2010). Trs85 directs a Ypt1 GEF, TRAPPIII, to the phagophore to promote autophagy. Proc. Natl. Acad. Sci. U S A 107, 7811–7816. 10.1073/pnas.1000063107 20375281 PMC2867920

[B39] Mackenzie I. R. Rademakers R. Neumann M. (2010). TDP-43 and FUS in amyotrophic lateral sclerosis and frontotemporal dementia. Lancet Neurol. 9, 995–1007. 10.1016/s1474-4422(10)70195-2 20864052

[B40] Majounie E. Renton A. E. Mok K. Dopper E. G. P. Waite A. Rollinson S.. (2012). Frequency of the C9orf72 hexanucleotide repeat expansion in patients with amyotrophic lateral sclerosis and frontotemporal dementia: a cross-sectional study. Lancet Neurol. 11, 323–330. 10.1016/S1474-4422(12)70043-1 22406228 PMC3322422

[B41] Malhotra V. (2013). Unconventional protein secretion: an evolving mechanism. EMBO J. 32, 1660–1664. 10.1038/emboj.2013.104 23665917 PMC3680731

[B42] Marat A. L. Dokainish H. McPherson P. S. (2011). DENN domain proteins: regulators of Rab GTPases. J. Biol. Chem. 286, 13791–13800. 10.1074/jbc.r110.217067 21330364 PMC3077579

[B43] Mata I. F. Jang Y. Kim C. H. Hanna D. S. Dorschner M. O. Samii A.. (2015). The RAB39B p.G192R mutation causes X-linked dominant Parkinson’s disease. Mol. Neurodegener. 10:50. 10.1186/s13024-015-0045-4 26399558 PMC4581468

[B44] May S. Hornburg D. Schludi M. H. Arzberger T. Rentzsch K. Schwenk B. M.. (2014). C9orf72 FTLD/ALS-associated Gly-Ala dipeptide repeat proteins cause neuronal toxicity and Unc119 sequestration. Acta Neuropathol. 128, 485–503. 10.1007/s00401-014-1329-4 25120191 PMC4159571

[B45] Mignogna M. L. Giannandrea M. Gurgone A. Fanelli F. Raimondi F. Mapelli L.. (2015). The intellectual disability protein RAB39B selectively regulates GluA2 trafficking to determine synaptic AMPAR composition. Nat. Commun. 6:6504. 10.1038/ncomms7504 25784538 PMC4383008

[B47] Mizielinska S. Grönke S. Niccoli T. Ridler C. E. Clayton E. L. Devoy A.. (2014). C9orf72 repeat expansions cause neurodegeneration in *Drosophila* through arginine-rich proteins. Science 345, 1192–1194. 10.1126/science.1256800 25103406 PMC4944841

[B46] Mizielinska S. Isaacs A. M. (2014). C9orf72 amyotrophic lateral sclerosis and frontotemporal dementia: gain or loss of function? Curr. Opin. Neurol. 27, 515–523. 10.1097/wco.0000000000000130 25188012 PMC4165481

[B48] Mori K. Weng S. M. Arzberger T. May S. Rentzsch K. Kremmer E.. (2013). The C9orf72 GGGGCC repeat is translated into aggregating dipeptide-repeat proteins in FTLD/ALS. Science 339, 1335–1338. 10.1126/science.1232927 23393093

[B49] Neary D. Snowden J. Mann D. (2005). Frontotemporal dementia. Lancet Neurol. 4, 771–780. 10.1016/S1474-4422(05)70223-4 16239184

[B50] Neumann M. Sampathu D. M. Kwong L. K. Truax A. C. Micsenyi M. C. Chou T. T.. (2006). Ubiquitinated TDP-43 in frontotemporal lobar degeneration and amyotrophic lateral sclerosis. Science 314, 130–133. 10.1126/science.1134108 17023659

[B51] Noda N. N. Fujioka Y. (2015). Atg1 family kinases in autophagy initiation. Cell. Mol. Life Sci. 72, 3083–3096. 10.1007/s00018-015-1917-z 25948417 PMC4506457

[B52] O’Rourke J. G. Bogdanik L. Yáñez A. Lall D. Wolf A. J. Muhammad A. K. M. G.. (2016). C9orf72 is required for proper macrophage and microglial function in mice. Science 351, 1324–1329. 10.1126/science.aaf1064 26989253 PMC5120541

[B53] Pearson J. P. Williams N. M. Majounie E. Waite A. Stott J. Newsway V.. (2011). Familial frontotemporal dementia with amyotrophic lateral sclerosis and a shared haplotype on chromosome 9p. J. Neurol. 258, 647–655. 10.1007/s00415-010-5815-x 21072532 PMC4696389

[B54] Peränen J. Auvinen P. Virta H. Wepf R. Simons K. (1996). Rab8 promotes polarized membrane transport through reorganization of actin and microtubules in fibroblasts. J. Cell Biol. 135, 153–167. 10.1083/jcb.135.1.153 8858170 PMC2121014

[B55] Pilli M. Arko-Mensah J. Ponpuak M. Roberts E. Master S. Mandell M. A.. (2012). TBK-1 promotes autophagy-mediated antimicrobial defense by controlling autophagosome maturation. Immunity 37, 223–234. 10.1016/j.immuni.2012.04.015 22921120 PMC3428731

[B56] Ponpuak M. Mandell M. A. Kimura T. Chauhan S. Cleyrat C. Deretic V. (2015). Secretory autophagy. Curr. Opin. Cell Biol. 35, 106–116. 10.1016/j.ceb.2015.04.016 25988755 PMC4529791

[B57] Renton A. E. Chiò A. Traynor B. J. (2014). State of play in amyotrophic lateral sclerosis genetics. Nat. Neurosci. 17, 17–23. 10.1038/nn.3584 24369373 PMC4544832

[B58] Renton A. E. Majounie E. Waite A. Simón-Sánchez J. Rollinson S. Gibbs J. R.. (2011). A hexanucleotide repeat expansion in C9ORF72 is the cause of chromosome 9p21-linked ALS-FTD. Neuron 72, 257–268. 10.1016/j.neuron.2011.09.010 21944779 PMC3200438

[B59] Riva N. Agosta F. Lunetta C. Filippi M. Quattrini A. (2016). Recent advances in amyotrophic lateral sclerosis. J. Neurol. 263, 1241–1254. 10.1007/s00415-016-8091-6 27025851 PMC4893385

[B60] Rohrer J. D. Isaacs A. M. Mizielinska S. Mead S. Lashley T. Wray S.. (2015). C9orf72 expansions in frontotemporal dementia and amyotrophic lateral sclerosis. Lancet Neurol. 14, 291–301. 10.1016/S1474-4422(14)70233-9 25638642

[B61] Rubino E. Rainero I. Chiò A. Rogaeva E. Galimberti D. Fenoglio P.. (2012). SQSTM1 mutations in frontotemporal lobar degeneration and amyotrophic lateral sclerosis. Neurology 79, 1556–1562. 10.1212/WNL.0b013e31826e25df 22972638 PMC3655323

[B62] Sato T. Iwano T. Kunii M. Matsuda S. Mizuguchi R. Jung Y.. (2014). Rab8a and Rab8b are essential for several apical transport pathways but insufficient for ciliogenesis. J. Cell Sci. 127, 422–431. 10.1242/jcs.136903 24213529 PMC3898603

[B63] Sellier C. Campanari M. L. Julie Corbier C. Gaucherot A. Kolb-Cheynel I. Oulad-Abdelghani M.. (2016). Loss of C9ORF72 impairs autophagy and synergizes with polyQ Ataxin-2 to induce motor neuron dysfunction and cell death. EMBO J. 35, 1276–1297. 10.15252/embj.201593350 27103069 PMC4910533

[B64] Seto S. Sugaya K. Tsujimura K. Nagata T. Horii T. Koide Y. (2013). Rab39a interacts with phosphatidylinositol 3-kinase and negatively regulates autophagy induced by lipopolysaccharide stimulation in macrophages. PLoS One 8:e83324. 10.1371/journal.pone.0083324 24349490 PMC3862771

[B65] Seto S. Tsujimura K. Koide Y. (2011). Rab GTPases regulating phagosome maturation are differentially recruited to mycobacterial phagosomes. Traffic 12, 407–420. 10.1111/j.1600-0854.2011.01165.x 21255211

[B66] Sreedharan J. Blair I. P. Tripathi V. B. Hu X. Vance C. Rogelj B.. (2008). TDP-43 mutations in familial and sporadic amyotrophic lateral sclerosis. Science 319, 1668–1672. 10.1126/science.1154584 18309045 PMC7116650

[B67] Stein J. L. Hibar D. P. Madsen S. K. Khamis M. McMahon K. L. de Zubicaray G. I.. (2011). Discovery and replication of dopamine-related gene effects on caudate volume in young and elderly populations (*N* = 1198) using genome-wide search. Mol. Psychiatry 16, 927–937, 881. 10.1038/mp.2011.32 21502949 PMC3140560

[B68] Sullivan P. M. Zhou X. Robins A. M. Paushter D. H. Kim D. Smolka M. B.. (2016). The ALS/FTLD associated protein C9orf72 associates with SMCR8 and WDR41 to regulate the autophagy-lysosome pathway. Acta Neuropathol. Commun. 4:51. 10.1186/s40478-016-0324-5 27193190 PMC4870812

[B69] Szatmári Z. Sass M. (2014). The autophagic roles of Rab small GTPases and their upstream regulators: a review. Autophagy 10, 1154–1166. 10.4161/auto.29395 24915298 PMC4203544

[B70] Therrien M. Rouleau G. A. Dion P. A. Parker J. A. (2013). Deletion of C9ORF72 results in motor neuron degeneration and stress sensitivity in *C. elegans*. PLoS One 8:e83450. 10.1371/journal.pone.0083450 24349511 PMC3861484

[B71] Thomas M. Alegre-Abarrategui J. Wade-Martins R. (2013). RNA dysfunction and aggrephagy at the centre of an amyotrophic lateral sclerosis/frontotemporal dementia disease continuum. Brain 136, 1345–1360. 10.1093/brain/awt030 23474849

[B72] Todd T. W. Petrucelli L. (2016). Insights into the pathogenic mechanisms of Chromosome 9 open reading frame 72 (C9orf72) repeat expansions. J. Neurochem. 138, 145–162. 10.1111/jnc.13623 27016280

[B73] Vance C. Al-Chalabi A. Ruddy D. Smith B. N. Hu X. Sreedharan J.. (2006). Familial amyotrophic lateral sclerosis with frontotemporal dementia is linked to a locus on chromosome 9p13.2–21.3. Brain. 129, 868–876. 10.1093/brain/awl030 16495328

[B75] Wang M. D. Gomes J. Cashman N. R. Little J. Krewski D. (2014). Intermediate CAG repeat expansion in the ATXN2 gene is a unique genetic risk factor for ALS—a systematic review and meta-analysis of observational studies. PLoS One 9:e105534. 10.1371/journal.pone.0105534 25148523 PMC4141758

[B74] Wang J. Menon S. Yamasaki A. Chou H. T. Walz T. Jiang Y.. (2013). Ypt1 recruits the Atg1 kinase to the preautophagosomal structure. Proc. Natl. Acad. Sci. U S A 110, 9800–9805. 10.1073/pnas.1302337110 23716696 PMC3683756

[B76] Webster C. P. Smith E. F. Bauer C. S. Moller A. Hautbergue G. M. Ferraiuolo L.. (2016). The C9orf72 protein interacts with Rab1A and the ULK1 complex to regulate initiation of autophagy. EMBO J. 35, 1656–1676. 10.15252/embj.201694401 27334615 PMC4969571

[B77] Weidberg H. Elazar Z. (2011). TBK1 mediates crosstalk between the innate immune response and autophagy. Sci. Signal. 4:pe39. 10.1126/scisignal.2002355 21868362

[B78] Wilson G. R. Sim J. C. H. McLean C. Giannandrea M. Galea C. A. Riseley J. R.. (2014). Mutations in RAB39B cause X-linked intellectual disability and early-onset Parkinson disease with α-synuclein pathology. Am. J. Hum. Genet. 95, 729–735. 10.1016/j.ajhg.2014.10.015 25434005 PMC4259921

[B79] Xi Z. Zinman L. Moreno D. Schymick J. Liang Y. Sato C.. (2013). Hypermethylation of the CpG island near the G4C2 repeat in ALS with a C9orf72 expansion. Am. J. Hum. Genet. 92, 981–989. 10.1016/j.ajhg.2013.04.017 23731538 PMC3675239

[B80] Xilouri M. Brekk O. R. Stefanis L. (2016). Autophagy and alpha-synuclein: relevance to Parkinson’s disease and related synucleopathies. Mov. Disord. 31, 178–192. 10.1002/mds.26477 26813776

[B81] Xu J. Fotouhi M. McPherson P. S. (2015). Phosphorylation of the exchange factor DENND3 by ULK in response to starvation activates Rab12 and induces autophagy. EMBO Rep. 16, 709–718. 10.15252/embr.201440006 25925668 PMC4467855

[B82] Yoshimura S. I. Egerer J. Fuchs E. Haas A. K. Barr F. A. (2007). Functional dissection of Rab GTPases involved in primary cilium formation. J. Cell Biol. 178, 363–369. 10.1083/jcb.200703047 17646400 PMC2064854

[B83] Zhang D. Iyer L. M. He F. Aravind L. (2012). Discovery of novel DENN proteins: implications for the evolution of Eukaryotic intracellular membrane structures and human disease. Front. Genet. 3:283. 10.3389/fgene.2012.00283 23248642 PMC3521125

